# Small Anellovirus in Hepatitis C Patients and Healthy Controls

**DOI:** 10.3201/eid1207.060234

**Published:** 2006-07

**Authors:** Elisabetta Andreoli, Fabrizio Maggi, Mauro Pistello, Silvia Meschi, Marialinda Vatteroni, Luca Ceccherini Nelli, Mauro Bendinelli

**Affiliations:** * University of Pisa, Pisa, Italy

**Keywords:** SAV, Hepatitis C, Genetic divergence, letter

**To the Editor:** Torquetenovirus (TTV) and torquetenominivirus (TTMV) are characterized by a small, negative-sense, circular, single-stranded DNA genome and by an extraordinary ability to produce chronic plasma viremia. Indeed, >80% of humans harbor variably high viral loads of TTV, TTMV, or both, in plasma, regardless of geographic provenance, age, sex, and health conditions (*1*). Currently, TTV and TTMV are classified as distinct species in the floating (although closely linked to the family *Circoviridae*) genus *Anellovirus*, but their extreme genetic heterogeneity and some distinctive features in genomic organization have led some to suggest that they should be classified as an independent family (*2**,**3*). Most recently, after examining serum specimens from patients with symptoms of an acute viral infection by using DNase sequence-independent single-primer amplification, Jones et al. (*4*) identified, among other viruses, 2 novel TTV- and TTMV-like agents. Because of their even smaller genomes (≈2.4 and 2.6 kb vs. 3.6–3.8 kb for TTV and 2.8–2.9 kb for TTMV), these agents were named small anelloviruses (SAVs).

Because tissue culture and serologic methods are not yet available, diagnosis of anellovirus infection relies exclusively on viral DNA detection. We tested 55 Italian hepatitis C patients (mean age 56 ± 14 years, male/female ratio 30/25, 53 TTV positive) and, for comparison, 35 healthy donors (mean age 36 ± 12 years, male/female ratio 17/18, 33 TTV positive) for SAV in plasma by using the polymerase chain reaction (PCR) primers described by Jones et al. (*4*), followed by direct amplicon sequencing. To increase assay sensitivity, a heminested PCR format was adopted that used a sense primer designed in a segment of the untranslated region that is highly conserved among all anelloviruses (5´-TCAAGGGGC AATTCGGGCT-3´). We found 5 positive results among the hepatitis C patients (9.1%, all of whom were TTV positive) and 3 positive results among healthy controls (8.6%); and all were confirmed by sequence data.

The amino acid sequences inferred from the coding segment of the amplicon of SAV in this study and the corresponding sequences of the 10 SAV in GenBank at the time of this writing were then aligned with representative TTV and TTMV sequences (Figure A1). This method allowed us to identify the motif WX_7_HX_3_CXCX_5_H, which is highly characteristic of the open reading frame 2 (ORF2) of anelloviruses (*5*), in all SAVs. SAV sequences, as well as a large number of TTVs and all TTMVs, were then used to construct a phylogenetic tree and to calculate the extent of genetic divergence within SAV, TTV, and TTMV. Although a precise phylogenetic description will require the analysis of full-length ORF2, the SAV sequences clustered quite separately from those of TTV and TTMV, and the extent of divergence observed among SAV was huge and in the same range as among TTV or TTMV. Furthermore, SAVs obtained from hepatitis C patients and healthy participants were intermingled (Figure A2).

While this study was under way, Biagini et al. reported a 12% prevalence of SAV viremia in French blood donors (*6*). Our results confirm the high prevalence of SAV viremia in healthy persons and extend the finding to hepatitis C patients. Our data, combined with those of Biagini et al., indicate that, since SAV clusters separately from previously identified anelloviruses, it should be considered a distinct species (or possibly genus). This would increase the already high genetic diversity of anelloviruses, further arguing for the appropriateness of creating a separate viral family.

Because the clinical and viral parameters of hepatitis C in SAV-positive patients were not significantly different from those in the SAV-negative patients (data not shown), our results suggest that, similar to TTV (*7*), SAV has little or no effect on the course of hepatitis C. Although anelloviruses have not yet been definitely linked to any specific disease, evidence is growing that they might be involved in acute respiratory diseases in children (*8**,**9*). Furthermore, a florid TTV replication in the respiratory tract correlated with severity of lung impairment in children with asthma (*10*). A precise appreciation of the wide range of viruses classified within the anelloviruses is a prerequisite to understanding such disease associations and the disease-inducing potential of these viruses in general.

## Figures and Tables

**Figure A1:**
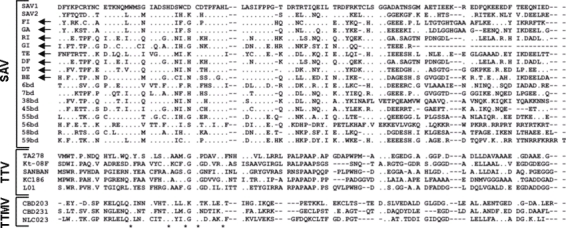
Amino acid sequences of the small anellovirus (SAV) obtained in the present study (arrows show accession nos. DQ409192 to DQ409199). GI, TE, and GA were from healthy donors; FI, RI, BE, DF, and DT were from hepatitis C patients. The sequences are aligned with all SAV present in GenBank at the time of writing and with representative torquetenovirus (TTV) and torquetenominivirus (TTMV) isolates. ClustalW alignments were manually edited and trimmed to eliminate poorly aligned positions and reduce homoplasy. As evaluated by Motif Scan and ProtParam analyses, the sequences were found to share similar properties (e.g., hydrophobicity profile, theoretical isoelectric point), irrespective of viral species. The WX_7_HX_3_CXCX_5_H motif highly characteristic of the open reading frame 2 of anelloviruses is indicated by asterisks.

**Figure A2:**
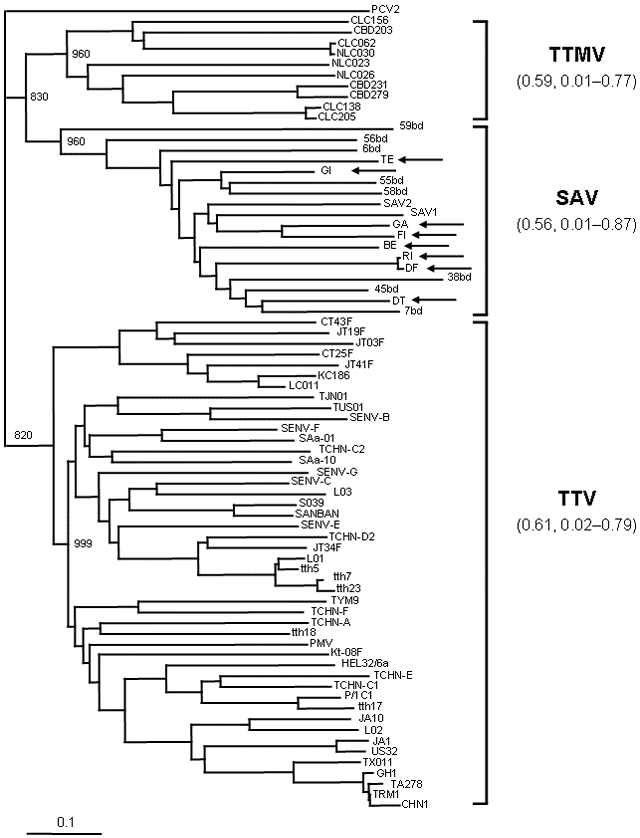
Amino acid sequences of the small anellovirus (SAV) obtained in the present study (arrows show accession nos. DQ409192 to DQ409199). GI, TE, and GA were from healthy donors; FI, RI, BE, DF, and DT were from hepatitis C patients. Evolutionary relationships between and within the 3 anelloviruses, torquetenovirus (TTV), torquetenominivirus (TTMV), and small anellovirus (SAV) are shown. Analysis is based on the 18 amino acid (aa) sequences of SAV above (75–104 aa), 46 open reading frame 2 (ORF2) amino acid sequences representative of the 5 TTV genogroups (96–104 aa), and 10 ORF2 amino acid sequences of TTMV (92–95 aa). Branching pattern was obtained by the FastME algorithm included in DAMBE software package (version 4.2.13). Bootstrap resampling was used to test the robustness of the tree, and bootstrap values >800 of 1,000 replicates are shown at the major branch points. The tree was drawn by the Treeview software (version 1.6.6). The ORF2 (110 aa) of porcine circovirus 2 was used as outgroup. Mean and range percentage amino acid distances within each of the 3 anelloviruses are shown in parentheses next to the virus names. Scale bar indicates the estimated number of amino acid substitutions per site.

## References

[1] Bendinelli M, Pistello M, Maggi F, Fornai C, Freer G, Vatteroni ML. Molecular properties, biology and clinical implications of TT virus, a recently identified widespread infectious agent of man. Clin Microbiol Rev. 2001;14:98–113. 10.1128/CMR.14.1.98-113.2001 11148004 PMC88963

[2] Hino S. TTV, a new human virus with single stranded circular DNA genome. Rev Med Virol. 2002;12:151–8. 10.1002/rmv.351 11987140

[3] Biagini P, Todd D, Bendinelli M, Hino S, Mankertz A, Mishiro S, Anellovirus. In: Fauquet CM, Mayo MA, Maniloff J, Desselberger U, Ball LA, editors. Virus taxonomy, 8th report of the International Committee for the Taxonomy of Viruses. New York: Elsevier/Academic Press; 2004. p. 335–41.

[4] Jones MS, Kapoor A, Lukashov VV, Simmonds P, Hecht F, Delwart E. New DNA viruses identified in patients with acute viral infection syndrome. J Virol. 2005;79:8230–6. 10.1128/JVI.79.13.8230-8236.2005 15956568 PMC1143717

[5] Takahashi K, Hijikata M, Samokhvalov EI, Mishiro S. Full or near full length nucleotide sequences of TT virus variants (types SANBAN and YONBAN) and the TT virus-like mini virus. Intervirology. 2000;43:119–23. 10.1159/000025034 10971131

[6] Biagini P, de Micco P, de Lamballerie X. Identification of a third member of the *Anellovirus* genus ("small anellovirus") in French blood donors. Arch Virol. 2006;151:405–8. 10.1007/s00705-005-0660-4 16328140

[7] Nishizawa Y, Tanaka E, Orr K, Rokuhara A, Ichijo T, Yoshizawa K, Clinical impact of genotype 1 TT virus infection in patients with chronic hepatitis C and response of TT virus to alpha-interferon. J Gastroenterol Hepatol. 2000;15:1292–7. 11129224

[8] Biagini P, Charrel RN, de Micco P, de Lamballerie X. Association of TT virus primary infection with rhinitis in a newborn. Clin Infect Dis. 2003;36:128–9. 10.1086/345552 12491220

[9] Maggi F, Pifferi M, Fornai C, Andreoli E, Tempestini E, Vatteroni ML, TT virus in the nasal secretions of children with acute respiratory diseases: relations to viremia and disease severity. J Virol. 2003;77:2418–25. 10.1128/JVI.77.4.2418-2425.2003 12551979 PMC141071

[10] Pifferi M, Maggi F, Andreoli E, Lanini L, De Marco E, Fornai C, Associations between nasal torquetenovirus load and spirometric indices in children with asthma. J Infect Dis. 2005;192:1141–8. 10.1086/444389 16136454

